# LAG3’s Enigmatic Mechanism of Action

**DOI:** 10.3389/fimmu.2020.615317

**Published:** 2021-01-08

**Authors:** Colin G. Graydon, Shifa Mohideen, Keith R. Fowke

**Affiliations:** ^1^ Department of Medical Microbiology and Infectious Diseases, University of Manitoba, Winnipeg, MB, Canada; ^2^ Department of Medical Micobiology, University of Nairobi, Nairobi, Kenya; ^3^ Department of Community Health Sciences, Max Rady College of Medicine, University of Manitoba, Winnipeg, MB, Canada; ^4^ Partners for Health and Development in Africa, Nairobi, Kenya

**Keywords:** LAG3, immune checkpoint, immune exhaustion, checkpoint inhibition, mechanism of action, Lymphocyte activation gene-3, immune checkpoint inhibitors

## Abstract

LAG3 is an important immune checkpoint with relevance in cancer, infectious disease and autoimmunity. However, despite LAG3’s role in immune exhaustion and the great potential of LAG3 inhibition as treatment, much remains unknown about its biology, particularly its mechanism of action. This review describes the knowns, unknowns and controversies surrounding LAG3. This includes examination of how LAG3 is regulated transcriptionally and post-translationally by endocytosis and proteolytic cleavage. We also discuss the interactions of LAG3 with its ligands and the purpose thereof. Finally, we review LAG3’s mechanism of action, including the roles of LAG3 intracellular motifs and the lack of a role for CD4 competition. Overall, understanding the biology of LAG3 can provide greater insight on LAG3 function, which may broaden the appreciation for LAG3’s role in disease and potentially aid in the development of targeted therapies.

## Introduction

Immune cells are chronically activated during cancer, chronic infection, autoimmune disease and graft versus host disease. In response to this persistent activation, immune cells become exhausted, losing the ability to produce cytokines or proliferate. This immune exhaustion is mediated, in part, by the expression of inhibitory co-receptors known as immune checkpoints (ICs). Immune exhaustion likely evolved to reduce the severity of autoimmune disease and deviant immune activation. However, when immune activation is a result of serious infection or cancer, a weaker immune response can be harmful. Inhibiting ICs can bolster the immune response against tumors and greatly improve survival in cancer patients (1). This translational potential has prompted a flurry of research into IC inhibition as treatment, such that our knowledge of IC biology and mechanism of action now lags. This is especially true for the IC–lymphocyte activation gene-3 (LAG3).

**Figure 1:**
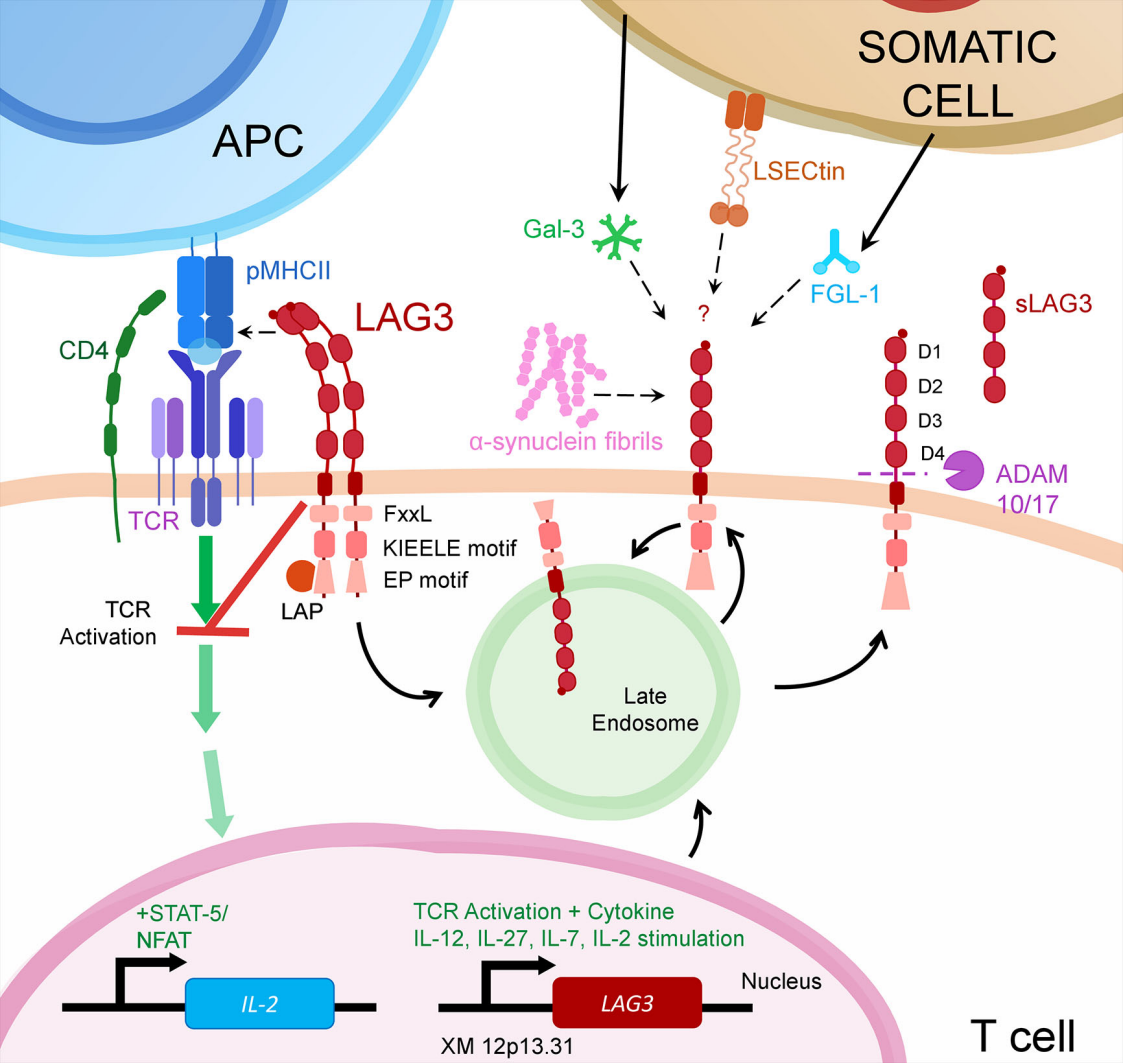
LAG3 transcription is upregulated following activation through the T cell Receptor (TCR) or from certain cytokines. Once translated, LAG3 resides in late endosomes for subsequent trafficking to the cell surface.There, LAG3 exists as an oligomer or monomer. While seemingly necessary for MHCII binding, LAG3 oligomerization may or may not be required for interaction with other LAG3 ligands (α-syn, Gal-3, L-SECtin or FGL-1). Upon ligand binding, LAG3 inhibits early steps of the TCR pathway in a manner dependent on LAG3’s cytoplasmic domain. This prevents activation of transcription factors, including NFAT, thereby decreasing cytokine production and proliferation. LAG3 surface expression is regulated by metalloproteases ADAM10/17 that proteolytically cleave LAG3, releasing it in soluble form (sLAG-3). LAG3 surface expression may also be regulated by endocytosis, which can be accelerated by ligand binding.

LAG3 is a member of the immunoglobulin superfamily and a CD4 ancestral homolog, resulting from a gene duplication event (2). Like CD4, LAG3 binds MHC class II (MHCII), but also FGL-1, α-synuclein fibrils (α-syn) and the lectins galectin-3 (Gal-3) and lymph node sinusoidal endothelial cell C-type lectin (LSECtin) (**Figure 1**) (3–6). As an immune checkpoint, LAG3 inhibits the activation of its host cell and generally promotes a more suppressive immune response. For example, on T cells, LAG3 reduces cytokine and granzyme production and proliferation while encouraging differentiation into T regulatory cells (7).

LAG3 is perhaps the third most advanced IC regarding clinical trial investigation, behind PD-1 and CTLA-4. Preliminary results of LAG3 blockade in melanoma and other cancers are very promising, particularly in combination with other IC blockade and/or for refractory cases (8). But despite the potential importance of LAG3 in immunotherapy, and the fact that LAG3 was identified in 1990, many knowledge gaps remain (listed in **Table 1**), perhaps the most glaring being its mechanisms of action. Understanding LAG3 mechanisms is important for identifying its yet unknown impacts and developing therapeutics that better inhibit or replicate LAG3 function. Here, we review mechanisms of LAG3 expression, ligand binding and function and identify major gaps in knowledge.

**Table 1:** Unknowns of LAG3 Biology.

	Description of knowledge gap
Expression	• What are LAG3 expression levels on innate T cells, B cells and pDCs during autoimmunity, cancer or infectious disease? • What regulates LAG3 endocytosis? • How does LAG3 RNA level correspond to surface expression? o Intracellular stores, endocytosis, and cleavage from cell surface complicate inferences of surface expression from RNA expression. • Does endocytosed LAG3 maintain function? • What % of intracellular LAG3 is *de novo* vs endocytosed?
Function	• What is the role of LAG3 on NK cells, B cells, pDCs and neurons? • Can LAG3 inhibit signaling through BCR, cytokine receptors, TLR? o Can blockade of LAG3 reverse these effects? • What is the impact of LAG3 blockade on innate T cells? • How does interaction with each of the five LAG3 ligands impact LAG3 activity? o What is the relative strength of each receptor? o Does this differ based on context? • Is sLAG3 non-functional? • Do LSECtin and Gal-3 bind to glycosylated regions of LAG3? • Does endocytosed LAG3 still function if bound to ligand in endosome?
Mechanism	• Is dimerization necessary to bind to all ligands? • Is dimerization necessary for LAG3 function? • Is LAG3 colocalization with the TCR necessary for LAG3 function? o If so, how do non-MHCII ligands facilitate this? • How does LAG3 inhibit activation following other stimuli (e.g., cytokines)? o Does it act through a similar pathway as for TCR stimulation? • What protein(s) bind to LAG3 intracellular domain to facilitate its function? o LAP is currently the only known interacting protein, but it cannot explain LAG3 function. Since ligand binding seems necessary for LAG3 function, it may be needed during screening for proteins that participate in LAG3 function through direct interaction. • What cytoplasmic motif(s) are involved in LAG3 function and what is their role? o Are different motifs involved in different aspects of LAG3 function? • If LAG3 inhibits activity of some kinase, as is currently suspected, what kinase does it inhibit and how? o Does LAG3 inhibit general activation by acting early in the pathway or does it act stronger on a particular pathway?

## Expression

### Cellular Distribution

LAG3 is mostly studied on conventional T cells and T regulatory cells, but is also expressed on unconventional T cells (i.e., γδT cells, mucosal-associated invariant T (MAIT) cells, invariant natural killer T (iNKT) cells), NK cells, B cells, plasmacytoid dendritic cells (pDCs) and neurons (5, 9–11). Innate T cells express very high levels of LAG3 after activation, implying a potentially important role on these cells (10, 12, 13). The impact of LAG3-targeted immunotherapy on these and non-T cells is largely unstudied but important, as it could significantly impact their function and therapeutic efficacy.

### Transcriptional Regulation

LAG3 expression is induced by activation through the TCR or by cytokines (particularly interleukin-12 (IL-12), IL-27, IL-15, IL-2, and IL-7) (14–19). LAG3 transcription is regulated by complex interactions with many possible regulatory and inducer elements including several potential transcription factor binding sites (16). Several transcription factors known to correlate with immune exhaustion, or induce expression of ICs also induce LAG3 expression, including TOX and NFAT (20, 21). Furthermore, LAG3 is inversely correlated with T-bet (a T-box transcription factor) (7, 22, 23). T-bet guides differentiation of cytotoxic T cells and has been reported as an important transcription factor in regulating immune exhaustion (22). Interestingly, the inverse correlation between LAG3 and T-bet is bidirectionally causal (i.e., deletion of either T-bet or LAG3 increases the expression of the other) on murine T cells, suggesting that LAG3 promotes exhaustion and vice-versa (7, 22, 23). Methylation, particularly of the LAG3 promoter, appears to tightly regulate LAG3 expression (24, 25).

### Soluble LAG3

As with other ICs, a soluble form of LAG3 is found in sera. Soluble ICs are produced by expression of splice variants or proteolytic cleavage of ICs, and can inhibit IC activity (e.g., soluble PD-1 occupies binding site on PD-L1/2) or maintain IC activity (e.g., soluble CTLA-4 competitively inhibits CD28 binding to CD80/86) (26). Three LAG3 splice variants are proposed to exist, two of which create a soluble form of the protein (27). However, the evidence for splice variants is limited. Instead, soluble LAG3 (sLAG3) is most likely from proteolytic cleavage of surface LAG3. Indeed, LAG3 is cleaved by a disintegrin and metalloproteinase (ADAM)10 and ADAM17 at the 20-aa connecting peptide between D4 and the transmembrane domain. *In vitro*, this cleavage results in a rise of sLAG3 in supernatants, implicating it as a major source of sLAG3 (28–30). T cells are not a major contributor to sLAG3, meaning other cells (especially pDCs) produce the bulk (29, 31). Some researchers have proposed that sLAG3 may function similarly to a synthetic LAG3 fusion protein (sLAG3-Ig), which binds to MHCII, thereby inhibiting the binding of LAG3 and its inhibitory function while inducing dendritic cell maturation (27). However, there is no published evidence of sLAG3 function nor its binding to MHCII (28). Instead, the purpose of cleavage is post-translational regulation of LAG3 as demonstrated in mice where non-cleavable LAG3 or metalloproteinase inhibitor impairs T cell immunity and in human cancer patients where a higher LAG3:ADAM10 ratio is associated with disease progression and poor prognosis (28, 30). Therefore, in regulating LAG3 surface expression, cleavage produces sLAG3 as a likely inert by-product.

### Intracellular Stores

Not all membrane embedded LAG3 resides on the cell surface. Even after cellular activation, approximately half of all LAG3 resides in late endosomes (32). Intracellular storage of LAG3 is thought to facilitate rapid trafficking to the cell surface (32), but may also represent previously surface expressed LAG3 that has been endocytosed. Endocytosis of LAG3 occurs following interaction with α-syn but has not been investigated for other LAG3 ligands (5). It is also unknown what proportion of intracellular LAG3 results from endocytosis versus *de novo* expression and whether LAG3 maintains inhibitory function following endocytosis. These factors complicate accurate assessment of surface expression through measurement of LAG3 transcript. Therefore, techniques that measure surface expression directly, such as flow cytometry or immunofluorescence, are preferred.

## Ligand Binding

### MHC Binding

LAG3 is a 70kDa transmembrane protein with four extracellular glycosylation sites. As a CD4 ancestral homolog, LAG3 shares similar structure but only 20% amino acid similarity (33). Like CD4, LAG3 is comprised of 4 extracellular domains, named D1-D4, with D4 located closest to the cell surface and D1 being most distal. Both D1 and D2 are necessary and sufficient for LAG3 binding to MHCII (34). While it is unclear how large the binding domain is, a 30 amino acid extra loop on the distal side of D1 is involved, perhaps containing the entire binding site. Point mutations in this region can enhance or reduce binding to MHCII, while deletion abolishes binding (34). Furthermore, this loop is the epitope for an antibody known to block the LAG3:MHCII interaction (clone 17B4) (6, 34).

Notably, mutations in another region of D1 also abolish MHCII binding. Huard et al. proposed that these mutations may prohibit MHCII binding by precluding oligomerization of LAG3, as these mutations disrupt even wild-type LAG3’s interaction with MHCII when co-expressed. It was later shown that LAG3 does indeed weakly oligomerize as a dimer and larger oligomer complex on the cell surface in a D1-dependent manner (29). Moreover, while dimeric sLAG3-Ig binds MHCII, monomeric sLAG3 cannot (28, 34–39). Together, this suggests that oligomerization is facilitated by a group of amino acids in D1, and that oligomerization is necessary for optimal LAG3 binding to MHCII.

Importantly, LAG3 does not bind all MHCII equally. Maruhashi et al. demonstrated that strong affinity of antigenic peptide for MHCII and expression of MHCII accessory molecules substantially increase LAG3 binding to MHCII. As the authors note, this indicates that LAG3 function is dependent on APC properties, the presented peptides and MHC haplotypes, bestowing a target selectivity that is unique among immune checkpoints (35, 40).

### Other Ligand Binding

It is important to note that while MHCII is a major ligand of LAG3, four other ligands have been discovered: FGL-1, Gal-3, LSECtin and α-syn. While α-syn’s impact on LAG3 inhibitory function remains unstudied, FGL-1, Gal-3 and LSECtin have each been shown to induce LAG3-mediated inhibition of T cell activation. Gal-3 and LSECtin are both lectins with carbohydrate-recognition domains (which may bind to glycosylated sites of LAG3) and oligomerization domains that facilitate LAG3 cross-linking (41, 42). Like MHCII, α-syn binds to the region of LAG3 D1 containing the extra loop and surrounding amino acids, with some dependence on D2, D3 or intracellular domain (5). Again, as with MHCII, LAG3 D1 and D2 are both necessary and sufficient for binding to the fibrinogen-like domain of FGL-1. However, the Y73F mutation in mice (Y77F in humans) that abolishes LAG3’s binding to MHCII, did not impact FGL-1 binding, illustrating that FGL-1 binds a different set of amino acids. This is also implied by the ability of the C9B7W antibody to block LAG3 interaction with FGL-1, but not MHCII.

## Mechanism of Action

### Functional Role of Ligand Binding

Once bound to its ligand, LAG3 can inhibit T cell activation. While engagement of LAG3 to its ligand is crucial for LAG3 function, the role of ligand interaction is unclear.

One role may be to bring LAG3 in proximity with the TCR. To support this role for ligand binding, the P111A mutation, that prevents LAG3 binding MHCII, does not lead to colocalization with the immune synapse (35); nor does T cell activation *via* unstable pMHCII, to which LAG3 is incapable of binding. Indeed, LAG3 ligation to non-cognate pMHCII during simultaneous activation by cognate pMHCI or unstable pMHCII, to which LAG3 does not bind, leads to a roughly two-third reduction in LAG3-mediated inhibition of IL-2 (35). In this model, LAG3 activity was lowest at higher peptide concentration. This may be explained as higher peptide concentration promoting clustering of pMHCI and TCR at the immune synapse, which crowds out LAG3 or reduces the ratio of LAG3 to signaling TCR complexes at the immune synapse. Further support for the necessity of LAG3 colocalization with the immune synapse to exert its function is given by a study that employs antibody-mediated cross-linking to activate T cells and engage LAG3. While cross-linking LAG3 and CD3 together inhibit T cell activation compared with CD3 cross-linking alone, cross-linking LAG3 and CD3 separately, but still simultaneously, does not (43). Other studies demonstrate that independent cross-linking of CD3 or TCR recruits LAG3 to the site of cross-linking (43, 44), which suggests that some colocalization may occur without ligand binding. However, the degree of colocalization may be insufficient for LAG3 activity. Together, these studies support the notion that LAG3 colocalization with the immune synapse is necessary for LAG3 function, and, therefore, may be the main role for LAG3 ligand binding.

The apparent importance of LAG3 colocalization with the immune synapse implies that LAG3 acts early in the TCR signaling pathway. This is further supported by a recent study that used a LAG3-expressing Jurkat T cell line to gain insight into the LAG3 mechanism. In these cells, LAG3 blockade significantly increased cytokine production and NFAT activity following activation. This effect was not impacted by PKCθ/δ inhibitor or stimulant, calcineurin inhibitor or a general kinase inhibitor, suggesting that LAG3 acts early in the TCR signaling cascade (27) and thereby further supporting the importance of LAG3 colocalization with the immune synapse.

However, it is not clear that binding of LAG3 to its alternative ligands would facilitate colocalization with the immune synapse, or how colocalization would inhibit activation by IL-7, as LAG3 has been shown to do (45). Therefore, while colocalization with the immune synapse may be important for inhibition of TCR signaling, this alone does not fully explain the role of LAG3 ligand binding.

Another role for ligand binding may be to enhance dimerization/oligomerization of LAG3, which may be necessary for LAG3 intracellular signaling. However, while this explanation is consistent with the literature, there is little evidence directly supporting this role, and thus remains speculative.

Overall, LAG3 ligand binding is crucial for its inhibitory function, potentially serving to colocalize LAG3 with signaling molecules and/or facilitate stable oligomerization.

### Competitive Inhibition of CD4 Is Not a Major Mechanism

Some have suggested that LAG3 inhibitory function is a result of competitive inhibition of CD4 due to its shared evolutionary origin and the fact that it binds MHCII with far greater affinity (35, 39, 40, 46). However, no evidence suggests LAG3 inhibits CD4-ligand interaction during T cell activation. In contrast, strong evidence shows that competitive inhibition of CD4 is not a major mechanism of LAG3-mediated inhibition.

### LAG3 Does Not Block CD4:MHCII interaction

One way to test for competition is by determining whether a LAG3 fusion protein blocks CD4 binding to MHCII. Using this method, one group found that LAG3 inhibits CD4 interaction with MHCII, but only when TCR is not engaged (39). The authors theorize that TCR engagement may either enhance CD4 affinity to MHCII, or change the binding to MHCII such that no competition with LAG3 occurs. A similar experimental model supported this assertion using three different cell lines (35).

### LAG3 Inhibits T Cells Independently of CD4

Furthermore, several studies have demonstrated that LAG3 relies completely on its intracellular domain for its inhibitory function (35, 47–49). Specifically, the existence of intracellular motifs that are necessary for LAG3 function (discussed below) implies a role for intracellular signaling and negates the necessity for receptor competition (46, 48–50). This is further supported by studies showing that the crosslinking of LAG3 induces LAG3 inhibitory function, even in the absence of MHCII (43, 51).

Further evidence of LAG3’s functional independence from CD4 is the existence of alternative LAG3 ligands that do not bind CD4 (3, 4, 42). Also, the fact that LAG3 inhibits activation of CD8^+^ T cells (4, 35, 52), on which it is expressed at higher levels than on CD4^+^ T cells, shows that LAG3 can function even in the absence of CD4.

### Intracellular Domain

When LAG3 binds its ligand, it transmits an inhibitory signal that prevents T cell activation, but how this signal is transmitted is largely unknown. Immune checkpoints typically inhibit T cell activation through cytoplasmic inhibitory domains such as ITIM or ITSM. These domains counteract TCR activation signals transmitted through kinases by recruiting phosphatases that contain SH2 binding domains to the immune synapse. LAG3 is unique among ICs in possessing no known inhibitory domain nor the C-X-X-C p56lck binding motif of CD4. Instead, the 54 amino acid long cytoplasmic domain of LAG3 has three regions that are conserved between mice and humans: a RRFSALE motif, a KIEELE motif and an EX/EP repeat motif on the C-terminus.

The KIEELE motif, in particular the lysine residue in this motif, was demonstrated as essential for LAG3-mediated inhibition in three murine studies, both *in vivo* and *in vitro*, by Workman et al. (47–49). However, the importance of this motif has not been replicated by others. In fact, one study found that deletion of the KIEELE motif had no impact on LAG3 function. Instead they found amino acids in the RRFSALE region were necessary (50). One may suspect that the serine residue in this region would be the most likely to play a role in LAG3 signaling, since serine phosphorylation can activate proteins. However, point mutation of this serine to alanine did not impact LAG3 function (47, 50). Instead, mutating either the phenylalanine or leucine (or both) to alanine in this region reduced LAG3-mediated inhibition of IL-2 production by half; the authors call this motif the FxxL motif. Pairing these mutations with deletion of the EX repeat motif (an expanded EP motif including part of the KIEELE motif) nearly doubled IL-2 production, seemingly turning LAG3 into a positive co-receptor (50). Interestingly, this behavior of LAG3 acting as a positive co-receptor has been shown before on CD4-3A9 cells with LAG3 lacking the cytoplasmic domain (47). It is unclear why the study by Maeda et al. did not show this positive co-receptor activity with the cytoplasmic domain deletion mutant. Interestingly, deletion of the EP or EX repeat motif alone does not impact LAG3 inhibition during MHCII engagement (47, 50)

The roles of FxxL or KIEELE motifs in LAG3 function remain uncharacterized; however, the existence of a 45kDa LAG3 associated protein (LAP), which directly binds the EP motif, invites speculation on its role. LAP shares 99.8% amino acid sequence identity with the C-terminal end of CENPJ, a protein mostly involved in centrosome and microtubule organization during cell division that can also augment STAT5 and NF-κB signaling (53, 54). This identity suggests it may serve in clustering LAG3 into lipid rafts or in trafficking of LAG3 from the microtubule organizing centre, where it is intracellularly localized in endosomes, to the cell surface; however, deletion of the EP motif does not impact LAG3 expression on the cell surface, nor does tubulin polymerization inhibitor, suggesting that LAG3 traffics through other means. One potential, yet unproven, role for the EP motif of LAG3 is to sequester LAP from participating in its potential coactivator roles for STAT5 and NF-kB, since LAG3 can inhibit STAT5 and Akt phosphorylation in response to IL-7 (45) or peptide (7) stimulation. This could help explain how LAG3 facilitates cell cycle arrest (13, 55, 56).

Overall, while LAG3 lacks classical inhibitory motifs, it nonetheless requires its intracellular domain to inhibit T cell activation. Regarding individual motifs, there is conflicting evidence on the importance of the KIEELE motif, one study demonstrating importance of the FxxL motif, and a consistently reported secondary role for the EP motif in LAG3 inhibitory function. It is important to note that to date, all analysis of LAG3 motifs and signaling have been performed on T cells activated with pMHCII, and mostly in mice. Greater attention to LAG3 function and mechanism on unconventional T cells, or other lymphocytes is warranted.

## Conclusion

Immune checkpoint blockade is revolutionizing the treatment of immunogenic cancers. While PD-1 and CTLA-4 are the only checkpoints approved for use in the clinic, antibody blockade of several others are in clinical trials. At the forefront of this line is LAG3. LAG3 blockade has demonstrated the ability to enhance the efficacy of PD-1 blockade in many models, including in a clinical trial (8). However, despite its incredible potential in treatment of cancer, autoimmunity, and infectious disease, much remains unknown about how LAG3 functions. Ultimately, determining LAG3’s mechanism of action and the impact of LAG3 blockade on cells other than conventional T cells would advance optimal use of LAG3 blockade and related therapies.

## Author Contributions

CG performed the literature review and wrote the first draft. SM performed the literature review and designed the figure. KF oversaw the project and provided final approval to the manuscript. All authors contributed to the article and approved the submitted version.

## Funding

This research was supported by Canadian Institutes of Health Research (CIHR) - funded Canadian HIV Cure Enterprise (CanCURE) Team Grant HB2 – 164064 and a CIHR operating grant PJT-148677. CG has been supported by Vanier scholarship from CIHR. SM is supported by Research Manitoba.

## Conflict of Interest

The authors declare that the research was conducted in the absence of any commercial or financial relationships that could be construed as a potential conflict of interest.
